# Unusual gastrointestinal histoplasmosis with jejunal perforation in a 57-year-old patient from Bogotá: Case report

**DOI:** 10.7705/biomedica.7959

**Published:** 2026-04-28

**Authors:** Diana-Marcela Gaviria-Delgado, Natalia Olaya, Laura Cristina Nocua-Báez

**Affiliations:** 1 Grupo Anatomopatología, Universidad Nacional de Colombia, Bogotá, D.C., Colombia Universidad Nacional de Colombia Universidad Nacional de Colombia Bogotá, D.C. Colombia; 2 Maestría en Ciencias - Microbiología, Facultad de Ciencias, Universidad Nacional de Colombia, Bogotá, D.C., Colombia Universidad Nacional de Colombia Facultad de Ciencias Universidad Nacional de Colombia Bogotá, D.C. Colombia; 3 Hospital Universitario Nacional de Colombia, Bogotá, D.C., Colombia Hospital Universitario Nacional de Colombia Hospital Universitario Nacional de Colombia Bogotá, D.C. Colombia; 4 Departamento de Patología, Facultad de Medicina, Universidad Nacional de Colombia, Bogotá, D.C., Colombia Universidad Nacional de Colombia Departamento de Patología, Facultad de Medicina Universidad Nacional de Colombia Bogotá, D.C. Colombia; 5 Especialidad en Infectología, Departamento de Medicina Interna, Facultad de Medicina, Universidad Nacional de Colombia, Bogotá, D.C., Colombia Universidad Nacional de Colombia Facultad de Medicina Universidad Nacional de Colombia Bogotá, D.C. Colombia

**Keywords:** Histoplasma, histoplasmosis, mycoses, delayed diagnosis, endemic diseases, granuloma, Colombia., histoplasma, histoplasmosis, micosis, diagnóstico tardío, enfermedades endémicas, granuloma, Colombia.

## Abstract

Histoplasmosis is an endemic fungal infection caused by *Histoplasma* species.

Disseminated forms, although rare, often involve the gastrointestinal tract and primarily affect immunocompromised patients. This article presents an unusual case of gastrointestinal histoplasmosis with jejunal perforation. A 57-year-old man presented with chronic abdominal pain, intermittent fever, cough, diaphoresis, nausea, and rapid weight loss.

Findings on computed tomography scans showed thickening and malignant lesions in the intestine, further evaluated through laparotomy, in addition to pulmonary involvement. Jejunal perforation and septic shock were identified and treated with antibiotics. The clinical course was unfavorable, resulting in patient’s death. Peritoneal liquid cultures and hemocultures were negative, but the biopsy histological analysis revealed the presence of intracellular yeasts compatible with *Histoplasma* spp.

This case highlights the importance of considering invasive fungal infections in apparently immunocompetent critically ill patients and reassessing diagnostic methods to ensure timely and effective treatment.

## Resumen

La histoplasmosis es una infección fúngica endémica causada por especies de *Histoplasma*. Las formas diseminadas, aunque raras, a menudo comprometen el tubo digestivo y afectan principalmente a pacientes inmunocomprometidos. En este trabajo se presenta un caso poco frecuente de histoplasmosis gastrointestinal con perforación del yeyuno. Un paciente de sexo masculino de 57 años presentó dolor abdominal crónico, fiebre intermitente, tos, diaforesis, náuseas y pérdida acelerada de peso.

Las tomografías computarizadas revelaron engrosamiento del intestino y lesiones malignas, posteriormente evaluadas mediante laparotomía, además de compromiso pulmonar. Se identificó perforación del yeyuno y choque séptico, el cual se trató con antibióticos. La evolución clínica fue desfavorable y el paciente falleció.

Los cultivos de líquido peritoneal y los hemocultivos fueron negativos, pero los análisis histológicos de la biopsia revelaron la presencia de levaduras intracelulares indicativas de *Histoplasma*. Este caso resalta la importancia de considerar las infecciones fúngicas invasivas en pacientes críticos aparentemente inmunocompetentes y revaluar los métodos de diagnóstico para lograr un tratamiento oportuno y eficaz.

Disseminated histoplasmosis is caused by the dysmorphic fungus belonging to the *Histoplasma* genus, prevalent in immunocompromised patients with HIV (human immunodeficiency virus), malignant hematologic diseases, or those undergoing cytotoxic therapy ^1^^,^^2^. Of those individuals affected by disseminated histoplasmosis, 70 to 90% experience gastrointestinal involvement and only a small fraction are symptomatic. Common symptoms include fever, weight loss, abdominal pain, and diarrhea, while severe cases may present with gastrointestinal bleeding, masses, or lesions ^3^^,^^4^.

Disseminated histoplasmosis occurs in 5 to 10% of cases, disseminating systemically to other organs, but is typically controlled by the immune system ^2^. The colon and ileum are the most frequently reported sites, although perforation of the jejunum is uncommon, with only three documented cases in the literature, according to Anderson *et al.*^4^^-^^6^. Except for the report by Goodwin *et al.,* all other cases involved HIV infection.

This article presents the case of a patient with gastrointestinal histoplasmosis with jejunal perforation, apparently immunocompetent. In Colombia, only one case of peritoneal histoplasmosis in an acquired immunodeficiency syndrome (AIDS) patient has been reported ^2^. However, gastrointestinal histoplasmosis with jejunal perforation has not yet been reported, making this the first documented case in the country.

## Case presentation

**Figure 1.**
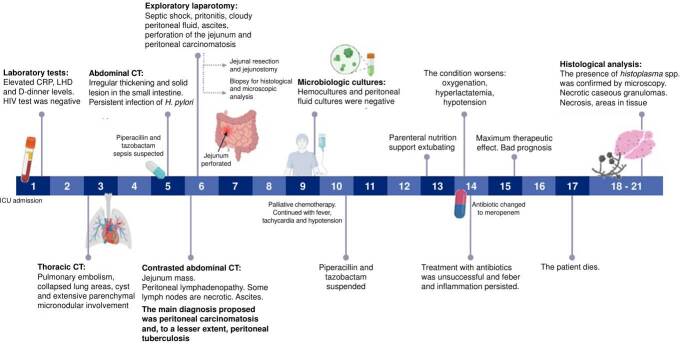
Timeline of clinical course of the patient. All the events occurred within a 21-day period.

A 57-year-old male patient, who lived in Bogotá and worked as a construction guard, presented to the medical service with complaints of abdominal pain, hyporexia, intermittent fever, diaphoresis, vomiting, difficulty in breathing, dry cough, and weight loss over three months (figure 1). He had antecedents of chronic gastritis treated with omeprazole (20 mg/12 h orally) and *Helicobacter pylori* infection, for which antibiotic treatment was completed.

Designed using bioRenderTM (https://www.biorender.com/)

CRP: C-reactive protein; CT: computed tomography; LDH: lactate dehydrogenase; HIV: human immunodeficiency virus; ICU: intensive care unit

The patient was normotensive (94/56 mm Hg), with a heart rate of 90 beats per minute, oxygen saturation of 90% with oxygen supplementation at 2 L/min, and was underweight (58 kg, 1.75 m).

Physical findings included palpable abnormal lymph nodes in the neck, decreased breath sounds in the right hemithorax on auscultation, dyspnea, and a distended, painful abdomen in the left flank. No evidence of leukocytosis, neutrophilia, or leukopenia was found.

Laboratory tests revealed elevated C-reactive protein (CRP), lactate dehydrogenase (LDH), and D-dimer levels (144 mg/L, 331 mg/dl, 5470 ng/ml, respectively; normal values: CRP < 10 mg/L; LDH < 0.8 mg/dl; D-dimer ≤ 500 ng/ml); moreover, the HIV test was negative.

A series of computed tomography (CT) scans were performed, too. Abdominal tomography revealed irregular thickening of a segment of the small intestine and a solid lesion in the mesentery, both on the left flank, along with multiple nodular images associated with the upper left parietal peritoneum, suggesting an infiltrative pattern. Retroperitoneal lymphadenopathy and ascites were also observed, and *H. pylori* infection was confirmed again in upper digestive tract endoscopy.

Pulmonary embolism was suspected due to elevated D-dimer levels as confirmed by thoracic computed tomography angiography, which showed areas of lung collapse, cysts, dilated bronchi, and extensive parenchymal micronodular involvement.

On the fifth day of admission, blood cultures were obtained on two separate occasions, with two bottles for aerobic bacteria and one bottle for anaerobic bacteria collected each time, but all remained negative. Moreover, intravenous piperacillin and tazobactam (4.5 g every 6 hours) were administered during 5 days for suspected bacteremia, pulmonary sepsis, and bacterial peritonitis; however, no response was observed.

Subsequent contrast-enhanced abdominal computed tomography revealed a lesion in the jejunum with perforation on its antimesenteric margin, accompanied by dense fluid in the abdominopelvic cavity and pneumoperitoneum. Thickening and retraction of the greater omentum, thickening of the peritoneum of the mesenteric leaves, and mucinous or necrotic adenomegalies were also observed.

**Figure 2.**
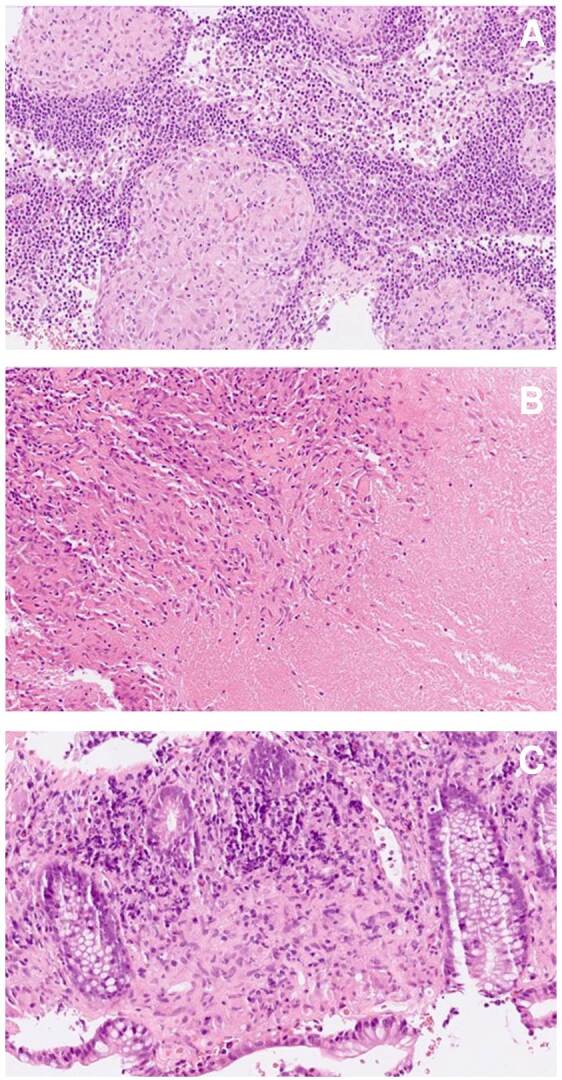
Peritoneal and intestinal pathology, hematoxilin and eosine, 100X. **A.** Granulomas in areas of chronic inflammation. B. Area of caseous necrosis with evident granular debris. **C.** Distorted colonic mucosa with dense and diffuse inflammatory infiltrate.

**Figure 3.**
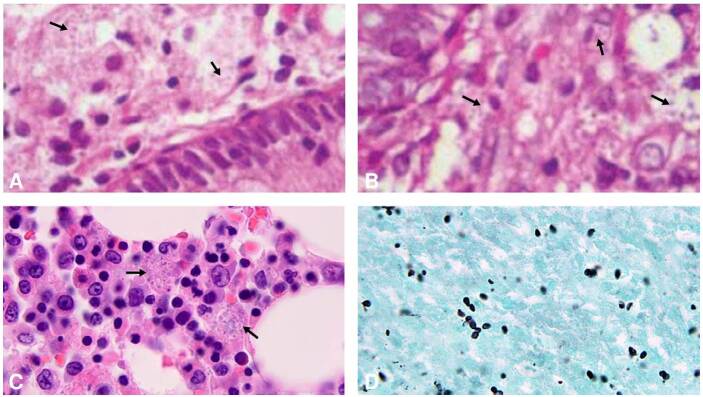
Detection of *Histoplasma* spp. infection in biopsy. **A.** The staining reveals the yeast as small diffuse purple dots indicated by the arrows due to cell overlay, hematoxilin and eosine, 400X. **B.** They are also observed in necrosis areas, hematoxilin and eosine, 400X. **C.** Presence of macrophages containing yeasts, hematoxilin and eosine, 400X. **D.** Presence of round or oval yeasts measuring between 4 and 6 μm, Grocott stain, 400X.

The patient underwent an exploratory laparotomy the following day, which confirmed a jejunal perforation, likely secondary to infiltration by a suspected transverse colon tumor. Intraoperative findings also included septic shock, generalized peritonitis, and turbid peritoneal fluid consistent with ascites and suggestive of peritoneal carcinomatosis originating from the transverse colon. Peritoneal fluid was collected for culture; the media used included blood culture bottles and direct plating on Sabouraud dextrose agar, incubated for three weeks, the result was negative. A segmental jejunal resection with jejunostomy was performed, and an omental biopsy was obtained for histopathological examination (figures 2 and 3). Both blood and peritoneal fluid cultures yielded negative results.

Given the assumption of carcinomatosis, palliative chemotherapy was administered from the ninth day of hospitalization. The patient’s condition continued to deteriorate, with persistent fever, tachycardia, and hypotension despite antibiotic treatment.

Intravenous meropenem (2 g every 8 hours) was escalated for sepsis management from day 14 until day 17, when the patient died, with no favorable clinical response observed. The short-term prognosis was poor, and the patient died after 17 days of hospitalization.

Microscopic analysis of the omental biopsy with hematoxylin and eosin staining revealed necrotizing caseous granulomas suggestive of yeast presence, confirmed by specific Grocott-Gomori methenamine silver staining, showing black oval yeasts characteristic of *Histoplasma* spp. (figure 3). The result of this test took two weeks; unfortunately, by the time it was available, the patient had already died.

## Ethical considerations

This report is a retrospective study conducted following all ethical considerations of Colombian regulation (Resolution 8430 of 1993, Article 11) and approved by the ethics committee of the university hospital of the *Universidad Nacional de Colombia*. The same committee made an exception to consent because the patient died during treatment before the manuscript was written. This exception is framed by judgment T_401/94 of the Constitutional Court of the Republic of Colombia: 1) when the patient’s mental state is abnormal; 2) when they are unconscious; 3) when the patient is a minor.

## Discussion

This report presents the case of a patient with persistent abdominal pain and fever, among other symptoms, caused by gastrointestinal histoplasmosis with perforation of the jejunum. This presentation of disseminated histoplasmosis is rare and sparsely reported in the literature. Computed tomography findings initially suggested possible peritoneal carcinomatosis, and to a lesser extent, peritoneal tuberculosis, instead of a fungal infection.

A similar case was reported in the literature: a 16-year-old girl whose histoplasmosis was not considered as the main diagnosis; instead, lymphoma or disseminated tuberculosis was suspected. Postmortem studies of lymph node biopsies confirmed the presence of *H. capsulatum* var. *capsulatum* yeasts ^7^.

Most cases of gastrointestinal histoplasmosis occur in male patients, as in our case; however, unlike our patient, they are frequently associated with an HIV diagnosis. In a large case series, South America was reported as the second most affected continent by this infection, after North America.

Although the most common histopathological finding in gastrointestinal involvement is ulceration of the colon or ileum, in our patient, the perforation occurred in the jejunum, with no macroscopic evidence of ulceration. The mortality rate reported among 119 patients was 6.7% ^8^.

More frequently, cases are described in patients with immunodeficiencies; however, several reports document events in apparently immunocompetent individuals, in whom the diagnostic challenge is greater due to the low initial suspicion of the disease. One such case occurred in México, involving a 29-year-old man who presented with gastrointestinal bleeding and hypovolemic shock, with the finding of a jejunal mass, peritoneal involvement, and colonic nodules that suggested a neoplastic etiology, similar to our case. This patient did receive treatment with amphotericin B; however, he ultimately died. Unfortunately, our patient did not receive treatment, as the diagnosis was established postmortem ^9^.

Unfortunately, fungal infections are underdiagnosed. Their symptoms are often insidious and common, leading to low clinical suspicion and underreporting ^10^^,^^11^. This is presumably due to a lack of clinical awareness, diagnostic tests, and misdiagnosis ^12^.

Clinically, peritoneal histoplasmosis and peritoneal tuberculosis are challenging to differentiate as their clinical manifestations are remarkably similar ^2^. Another potential infectious diagnosis is gastrointestinal or disseminated cryptococcosis, which has a similar presentation and can also affect immunocompetent patients, particularly with the *Cryptococcus gattii* species ^13^. Furthermore, mucosal lesions in gastrointestinal histoplasmosis may be mistaken for carcinomas ^4^.

The omental biopsy revealed the presence of necrotizing caseous granulomas (figure 2). The frequency of granulomas was reported in 39.3% of HIV-positive patients reviewed in the literature by Assi *et al*. (2006) and in 44.4% of the patients evaluated in their study. However, patients with advanced cellular immunodeficiency may not present the typical characteristics of granulomatous inflammation and may not be observable ^4^. These granulomas are also common in peritoneal tuberculosis, but histologically, distinguishing them from gastrointestinal histoplasmosis is not possible ^2^.

Peritoneal fluid culture and hemoculture were negative; additionally, there was no response to antibiotic treatment. Although fungal infection was not initially suspected, it was later confirmed by microscopy. It is worth mentioning that hemoculture has limitations in terms of time and sensitivity, leading to possible false negatives, diagnostic delays, and increased mortality ^2^^,^^4^.

A systematic review showed 76.71% sensitivity for cultures of samples from different tissues and from blood ^14^. It has also been reported that peritoneal fluid cultures may be negative in cases of peritoneal histoplasmosis ^2^.

Histoplasma antigen and molecular tests have been suggested as the methods of choice for diagnosis due to their better performance, with 95.48% and 95.35% sensitivity, respectively ^15^. Histoplasma antigen, particularly when using the lateral flow assay, can be detected in either urine or blood, with higher sensitivity observed in urine (92% in urine vs. 82% in blood).

In the context of gastrointestinal involvement, as in the present case, diagnosis is generally established through histopathological examination. In a descriptive review of 123 cases, 88.6% were diagnosed using this method. This is mainly due to the nonspecific nature of the disease’s clinical manifestations, with abdominal pain being the most frequently reported symptom. In most cases of gastrointestinal histoplasmosis, the colon is the predominant site of involvement; in our case, however, the jejunum was affected -an uncommon location-^8^^,^^16^^,^^17^. Polymerase chain reaction is another diagnostic test for histoplasmosis with excellent performance, demonstrating 100% sensitivity and 95% specificity. It also enables a rapid diagnosis; however, its accessibility is more limited, primarily due to constraints in availability and cost ^18^.

For patients with unexplained gastrointestinal pathologies or HIV positive, it is recommended to perform fungal stains and microbiological cultures in search of these rare infectious etiologies ^4^. This is more relevant in places considered endemic for infectious diseases.

Histoplasmosis is an endemic mycosis in the Colombian Andean region ^19^. In this patient, construction work was a risk factor to consider, as he may have been exposed to fungal spores at work ^20^. Similarly, a case of peritoneal histoplasmosis was reported in a patient who worked as a heavy machinery operator in Bogotá ^2^.

Histoplasmosis is a growing public health concern. There is a high frequency of underdiagnosis, and it is often misdiagnosed, confusing it with pathologies such as neoplastic diseases^21^^-^^23^. This leads to inadequate treatment and an increase in the mortality rate, which reaches 80% when treatment is late ^3^.

It is essential to reconsider when to suspect the disease, especially in rare manifestations, to make a faster diagnosis using methods with reliable performance. Incorporating molecular biology and antigen techniques with conventional methods could speed up the diagnosis and allow more effective management strategies.

This case underscores the importance of considering histoplasmosis in patients presenting with a subacute gastrointestinal syndrome accompanied by systemic and constitutional symptoms, as a differential diagnosis in the context of findings suggesting a neoplastic etiology. Early recognition should prompt timely diagnostic testing for *Histoplasma*, particularly urinary antigen detection using the lateral flow assay, which is a rapid and high-performance method. This is especially relevant given that histopathological diagnosis often involves considerable delays ^8^.
